# *Hippocampuswhitei* Bleeker, 1855, a senior synonym of the southern Queensland seahorse *H.procerus* Kuiter, 2001: molecular and morphological evidence (Teleostei, Syngnathidae)

**DOI:** 10.3897/zookeys.824.30921

**Published:** 2019-02-14

**Authors:** Graham Short, David Harasti, Healy Hamilton

**Affiliations:** 1 California Academy of Sciences, San Francisco, USA California Academy of Sciences San Francisco United States of America; 2 Fisheries Research, Port Stephens Fisheries Institute, New South Wales, Australia Fisheries Research, Port Stephens Fisheries Institute Port Stephens Australia; 3 NatureServe, Arlington, Virginia, USA NatureServe Arlington United States of America

**Keywords:** Acanthomorpha, Australia, COI, marine fish, morphology, systematics, taxonomy

## Abstract

The taxonomic status of the seahorse *Hippocampusprocerus* Kuiter, 2001, type locality Hervey Bay, QLD, Australia, was re-examined based on its strong morphological similarity and geographical proximity to its congener *H.whitei* Bleeker, 1855, a species recorded in ten estuaries of New South Wales, Australia. Kuiter (2001) distinguished *H.procerus* from *H.whitei* by a taller coronet, marginally lower meristics, and spinier physiognomy. Meristic, morphometric, and key diagnostic morphological character comparisons from vouchered specimens of the two purported species collected from Sydney Harbour, Nelson Bay, Port Stephens, NSW and Hervey Bay, Bundaberg, and Moreton Bay, QLD did not show diagnostic differences to support species-level classification of *H.procerus*. Furthermore, partial mitochondrial COI sequence data from specimens sampled from known geographical distributions in NSW and Southport, QLD failed to discriminate between populations as a result of shared haplotypes, and revealed an average intraspecific divergence of 0.002%. *Hippocampusprocerus* is hereby placed in the synonymy of *H.whitei*; a redescription is provided, with a revised record of its range across eastern Australia.

“*Sea-horse, or Hippocampus. This animal, like the Flying-fish, being commonly known, a description is not necessary. It is the Syngnathus Hippocampus of Linnaeus. See plate 264*” (White 1790: 295).

## Introduction

*Hippocampuswhitei* Bleeker, 1855, is a geographically restricted species of seahorse recorded in ten coastal estuaries and embayments of central New South Wales (NSW), and also farther north in the Tweed River, Australia. It can be found occurring in a variety of habitats including seagrasses, soft corals, sponge gardens, and artificial structures to depths of 15 m (Vincent et al. 2004; Hellyer et al. 2011; Harasti et al. 2014a). It is listed as ‘Endangered’ under criterion A2bc of the IUCN Red List due its restricted distribution, loss of essential marine habitats, and associated population declines in developed urban estuaries, including Port Stephens and Sydney Harbour (Harasti 2016; Harasti and Pollom 2017). Therefore, the conservation of *H.whitei* populations through the implementation of species monitoring and various management options, such as habitat protection and no-take policies are important for their protection and recovery, as well as for scientific, ecological, and economic purposes (Harasti et al. 2010, 2012, 2014b; Hellyer et al. 2011; Vincent et al. 2011).

Efforts to advance the conservation of seahorse populations are highly dependent on being able to confidently identify individual species in and beyond their known geographic distributions. The most recent and comprehensive taxonomic review of the genus *Hippocampus* (Lourie et al. 2016) places the number of recognized seahorse taxa occurring in Australia at sixteen species. However, the validity of several putative species remains uncertain. Seahorses are challenging to identify: multiple species have been synonymized based on recent genetic data, and there are many previous descriptions now recognized as spurious misidentifications attributed to the use of unreliable or non-diagnostic morphological characters (Lourie et al. 2016).

The taxonomic identity of *Hippocampusprocerus* Kuiter, 2001, originally described from Hervey Bay, Queensland (QLD), with a known distribution in Gold Coast Seaway and Moreton Bay, QLD, has been in question due to its strong morphological similarity and geographic proximity to *H.whitei* (Lourie et al. 2016). It was distinguished from *H.whitei* primarily by a taller coronet, subtle differences in meristic characters, and a spinier physiognomy (Kuiter 2001). These indistinct morphological differences between the two species prompted a re-examination of the holotype and non-type specimens of *H.procerus* from Hervey Bay, paratypes of *H.procerus* from Bundaberg and Moreton Bay, QLD, non-type specimens of *H.procerus* from Mackay, Elliot Heads, and Gold Coast Seaway, QLD, and non-type specimens of *H.whitei* from Sydney Harbour and Nelson Bay, Port Stephens, NSW, employing meristic, morphometric, and key diagnostic morphological character comparisons. The diagnostic characters comprise in part the absence or presence of principal spines, including snout, cleithral ring, neck, and subdorsal spines, with respect to their spatial position on the head and body. We demonstrated that the morphological characters in the non-type specimens of *H.whitei* corresponded closely with the examined non-type specimens, paratypes, and the holotype specimen of *H.procerus*, including: coronet height, absence of neck spines, indiscernible or small parietal spine, the numbers and positions of cleithral ring and subdorsal ridge spines, and overall spine physiognomy. Partial mitochondrial COI sequence data generated from specimens sampled from known geographical distributions in NSW and from Southport, QLD failed to discriminate between populations as a result of shared haplotypes, and revealed an average intraspecific divergence of 0.002%. *Hippocampuswhitei* Bleeker, 1855, is herein formally redescribed as a senior synonym of *H.procerus*. This estuarine species is apparently endemic to estuaries of central NSW, the Tweed River, and southern QLD.

## Materials and methods

**Figure 1.**
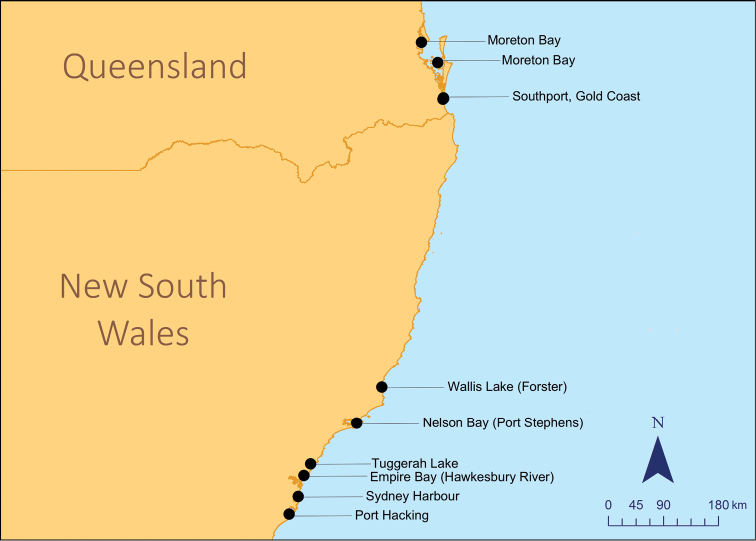
Collection locations for *H.procerus* in QLD and *H.whitei* in NSW, Australia.

Four individuals referred to as *H.procerus*, based on known locality of this species (Kuiter 2001), were collected from Southport, Gold Coast Harbour, QLD in 2014 by seine in seagrass beds in 1–2 m depth or by hand nets while scuba diving in less than 8 m depth (Figure 1), from which tissue was sampled from the caudal tip of the tails and preserved in a NaCl-saturated DMSO solution for genetic analyses. Similarly, thirty-one individuals of *H.whitei* were tissue sampled from the caudal tip of the tail at seven localities along the species’ known geographic range in New South Wales, Australia (Table 1, Figure 1) from 2007–2009. DNA extraction, PCR amplification, alignment, and analysis of partial mitochondrial cytochrome c oxidase subunit I (COI) sequences was performed following standard protocols described in Hamilton et al. (2017). Genetic distances (uncorrected *p*-distances) were calculated and neighbour-joining (NJ) trees constructed with confidence levels assessed using 1000 bootstrap replications based on partial COI using MEGA v. 7.0.26 (Kumar et al. 2017).

**Table 1.** List of *H.whitei* specimens, and those referred to as *H.procerus*, including collection locality, voucher or field number, and COI GenBank accession numbers.

	Species	Locality	Voucher / Field	COI GenBank accession no.
1	* Hippocampus procerus *	Southport, QLD, Australia	CAS 241511	MH745371
2	* Hippocampus procerus *	Southport, QLD, Australia	CAS 241512	MH745372
3	* Hippocampus procerus *	Southport, QLD, Australia	CAS 241513	MH745373
4	* Hippocampus procerus *	Southport, QLD, Australia	CAS 241514	MH745374
5	* Hippocampus whitei *	Sydney, NSW, Australia	HH-0418	MH745375
6	* Hippocampus whitei *	Sydney, NSW, Australia	HH-0419	MH745376
7	* Hippocampus whitei *	Sydney, NSW, Australia	HH-0469	MH745377
8	* Hippocampus whitei *	Sydney, NSW, Australia	HH-0470	MH745378
9	* Hippocampus whitei *	Sydney, NSW, Australia	HH-0667	MH745379
10	* Hippocampus whitei *	Empire Bay, NSW, Australia	HH-1276	MH745380
11	* Hippocampus whitei *	Empire Bay, NSW, Australia	HH-1277	MH745381
12	* Hippocampus whitei *	Nelson Bay, NSW, Australia	HH-1287	MH745382
13	* Hippocampus whitei *	Tuggerah Lake, NSW, Australia	HH-1290	MH745383
14	* Hippocampus whitei *	Tuggerah Lake, NSW, Australia	HH-1291	MH745384
15	* Hippocampus whitei *	Tuggerah Lake, NSW, Australia	HH-1292	MH745385
16	* Hippocampus whitei *	Nelson Bay, NSW, Australia	HH-1295	MH745386
17	* Hippocampus whitei *	Nelson Bay, NSW, Australia	HH-1299	MH745387
18	* Hippocampus whitei *	Nelson Bay, NSW, Australia	HH-1300	MH745388
19	* Hippocampus whitei *	Nelson Bay, NSW, Australia	HH-1305	MH745389
20	* Hippocampus whitei *	Port Hacking, NSW, Australia	HH-1321	MH745390
21	* Hippocampus whitei *	Port Hacking, NSW, Australia	HH-1322	MH745391
22	* Hippocampus whitei *	Port Hacking, NSW, Australia	HH-1329	MH745392
23	* Hippocampus whitei *	Port Hacking, NSW, Australia	HH-1330	MH745393
24	* Hippocampus whitei *	Port Hacking, NSW, Australia	HH-1340	MH745394
25	* Hippocampus whitei *	Port Hacking, NSW, Australia	HH-1341	MH745395
26	* Hippocampus whitei *	Nelson Bay, NSW, Australia	HH-1352	MH745396
27	* Hippocampus whitei *	Nelson Bay, NSW, Australia	HH-1353	MH745397
28	* Hippocampus whitei *	Nelson Bay, NSW, Australia	HH-1354	MH745398
28	* Hippocampus whitei *	Nelson Bay, NSW, Australia	HH-1357	MH745399
30	* Hippocampus whitei *	Nelson Bay, NSW, Australia	HH-1359	MH745400
31	* Hippocampus whitei *	Forster, NSW, Australia	HH-1363	MH745401
32	* Hippocampus whitei *	Nelson Bay, NSW, Australia	HH-1364	MH745402
33	* Hippocampus whitei *	Nelson Bay, NSW, Australia	HH-1365	MH745403
34	* Hippocampus whitei *	Forster, NSW, Australia	HH-1366	MH745404
35	* Hippocampus whitei *	Forster, NSW, Australia	HH-1367	MH745405
36	* Hippocampus whitei *	Forster, NSW, Australia	HH-1368	MH745406

Proportional measurements and counts based on eight morphometric and six meristic variables (Tables 2, 3), including 17 diagnostic morphological characters, were performed on dried or ethanol-preserved specimens and high-resolution digital images of specimens using ImageJ (Rasband et al. 1997) to the nearest 0.1 mm following Lourie and Randall (2003) and Lourie and Kuiter (2008). External morphological characters were documented using a dissecting microscope or on high-resolution digital images of specimens. The holotype specimen of *H.whitei* is unknown. The original description of *H.whitei* Bleeker, 1855 is based on an artistic and non-informative rendering (White 1790: 264, plate 50) from Sydney Harbour, NSW. Morphometric measurements were recorded for two non-type specimens of *H.whitei* from Nelson Bay, NSW, two non-type specimens of *H.procerus* from Southport, Gold Coast Harbour, QLD, two non-type specimens of *H.procerus* from Moreton Bay, QLD, and one non-type specimen of *H.procerus* from Bundaberg and Mackay, QLD, respectively (Table 2). These data were compared to morphometric data from the holotype specimen of *H.procerus* from Hervey Bay, QLD (Table 2). Meristic counts and diagnostic morphological characters were recorded for 12 non-type specimens of *H.whitei*, including two from Sydney Harbour, six from Pittwater, and four from Nelson Bay, NSW, and 13 type and non-type specimens of *H.procerus*, including four from Southport, Gold Coast Harbour, QLD, two from Moreton Bay, one from Hervey Bay, QLD, one from Elliot Bay, four from Bundaberg, and one from Mackay, QLD (Table 3). These data were compared with similar morphological data (Table 3) for the holotype specimen of *H.procerus* from Hervey Bay, QLD.

**Table 2.** Counts and morphometric measurements of specimens of *H.whitei* from Nelson Bay, NSW and those referred to as *H.procerus* from Southport, Moreton Bay, and Hervey Bay, QLD. Abbreviations: SnD (snout depth), SnL (snout length), CH (coronet height), HL (head length), HD (head depth), PO (post-orbital length), TrL (trunk length), TaL (tail length), SL (standard length). Numbers separated by a colon represent proportions (%). Lines present, from top to bottom, counts for trunk rings, tail rings, subdorsal rings, dorsal and pectoral fin rays.

	* H. whitei *	* H. whitei *	* H. procerus *	* H. procerus *	* H. procerus *	* H. procerus *	* H. procerus *	* H. procerus *	* H. procerus *
Voucher or field number	PSFC-DH-1	PSFC-DH-2	CAS 241511	CAS 241512	QM I.30772	AMS I.12554	AMS E2914	CAS-SU 36420-3	CAS-ICH 13406
Type status	non-type	non-type	non-type	non-type	Paratype	Paratype	Holotype	non-type	non-type
Sex	adult male	subadult female	adult female	juvenile female	subadult female	adult male	adult female	subadult female	adult male
Location	Nelson Bay, NSW	Nelson Bay, NSW	Southport, QLD	Southport, QLD	Morerton Bay, QLD	Morerton Bay, QLD	Hervey Bay, QLD	Bundaberg, QLD	Mackay, QLD
Trunk rings	11	11	11	11	11	11	11	11	11
Tail rings	34	34	35	35	34	35	35	35	35
Subdorsal rings	3	3	3	3	3	3	3	3	3
Dorsal fin rays	17	17	18	18	18	18	18	18	18
Pectoral fin rays	16	16	16	16	16	16	18	17	18
SL (mm)	142.7	47.7	122.1	58.5	95.0	105.0	123.3	124.7	113.4
SnD:SnL	23.9	24.9	24.2	27.6	28.7	23.9	31.2	21.9	23.5
CH:HL	45.5	46.5	45.1	47.8	52.9	48.9	44.8	50.8	46.7
HD:HL	57.0	60.6	55.6	60.4	48.4	52.4	47.8	47.0	49.5
SnL:HL	46.2	45.5	43.6	46.2	49.3	44.3	43.6	48.9	46.1
PO:HL	33.8	37.0	40.6	38.1	38.9	36.7	36.8	34.9	33.8
HL:SL	22.9	20.3	21.5	24.2	27.3	25.7	25.2	22.6	21.9
TrL:SL	41.4	29.3	33.8	39.1	45.7	37.6	39.3	31.8	38.7
TaL:SL	58.6	50.5	66.2	60.9	63.8	62.5	60.7	65.26	54.8

**Table 3.** Comparison of diagnostic morphological characters in non-types specimens of *H.whitei* from NSW and non-type and type specimens of *H.procerus* from QLD.

	* H. whitei *	* H. whitei *	* H. whitei *	* H. procerus *	* H. procerus *	* H. procerus *	* H. procerus *	* H. procerus *	* H. procerus *	* H. procerus *
Voucher number	CAS-SU 36407	CAS-SU 36417	PSFC-DH	CAS 24151- 14	QM I.30772	AMS I.12554	AMS E2914	QM I.39230	CAS-SU 36420-2,3,4	CAS-ICH 13406-1
Type status	non-type	non-type	non-type	non-type	Paratype	Paratype	Holotype	non-type	Paratype	non-type
Location	Port Jackson, NSW	Port Hacking, NSW	Nelson Bay, NSW	Southport, QLD	Moreton Bay, QLD	Moreton Bay, QLD	Hervey Bay, QLD	Elliot Heads, QLD	Bundaberg, QLD	Mackay, QLD
Number of specimens	2	6	4	4	1	1	1	1	4	1
Coronet	distinct and tall									
Neck spines	absent								absent/present	absent
Upper cleithral spine	present									
Mid cleithral spine	present									
Ventral cleithral spine	present (single or double)			present (single)						
Upper cleithral spine position	near top of pectoral fin base									
Mid cleithral spine position	near bottom of pectoral fin base									
Ventral cleithral spine position	ventral extent of head									
Subdorsal rings spines	3/0,1,0	3/0,1,0	3/0,1,0	3/0,1,0	3/0,1,0	3/0,1,0	3/0,1,0	3/0,1,0	3/0,1,0	3/0,1,0
Parietal spine	absent or blunt	absent or blunt	absent or blunt	absent or blunt	absent or blunt	absent or blunt	absent or blunt	absent or blunt	absent/blunt/present	present
Lateral head spine	present									
Snout spine	present									
Dorsal eye spine	present (single)	present (one paratype double)	present (single)							
Small posterior eye spine	present									
Superior trunk ridge spines enlarged	1,8	1,8	1,8	1,8	1,8	1,7,8,9	1,7,8,9	1,8	1,8 - 1,7,8,9	1,4,6,7,8,9
Lateral trunk ridge spines enlarged	8–11	8–11	8–11	8–11	8–11	8–11	2–11	6–11	2–11	4–11
Inferior trunk ridge spines enlarged	4–11	4–11	4–11	4–11	4–11	4–11	4–11	5–11	5–11	5–11
Superior tail ridge spines enlarged	1–12	1–12	1–12	1–12	1–12	1–12	1–13	1–10	1–10	1–12
Inferior tail ridge spines enlarged	1–8	1–8	1–8	1–8	1–8	1–9	1–7	1–10	1–10	1–5

## Taxonomy

### Hippocampus whitei

Taxon classificationAnimaliaSyngnathiformesSyngnathidae

Bleeker, 1855

Figures 2 , 3 , 4 , 5 , 6 , 7 , 8 , 9 , 10 , 11 , 12 , Tables 1 , 2 , 3 , 4 , 5 , 6 Common names: White’s seahorse; New Holland seahorse; Sydney seahorse (Australia)

Hippocampus novaehollandiae Steindachner, 1866: 474 (Sydney Harbour, Australia).
Hippocampus procerus Kuiter, 2001: 328–329, figs. 4, 40 (Hervey Bay, Queensland, Australia).

#### Material examined.

CAS 241511, adult female, Wave Island, Southport, QLD, Australia, 27°55'56.2"S 153°25'08.4"E, 5 m depth, in seagrass bed, November 27, 2014; CAS 241512, juvenile female, South West Wall, Southport, QLD, Australia, 27°56'32.7"S 153°25'14.7"E, 5 m depth, rocks and sand, November 26, 2014; CAS 241513, adult male, South West Wall, Southport, QLD, Australia, 27°56'32.7"S 153°25'14.7"E, 5 m depth, rocks and sand, November 26, 2014; CAS 241514, subadult male, Broadwater, QLD, Australia, 27°57'09.3"S 153°24'37.0"E, in seagrass bed, November 27, 2014; PSFC-DH (Port Stephens Fisheries Centre NSW field designation), 4 specimens in lot, PSFC-DH-1 adult male, PSFC-DH-2, subadult female, Nelson Bay, NSW, Australia, 32°42'59.9"S 152°08'57.2"E, 7 m depth, sandy rubble and seagrass, 2007-2016; SU 36407, 2 specimens in lot, adult males, Port Jackson, NSW, Australia, 33°50'42.6"S 151°14'50.5"E; SU 36417, 6 specimens in lot, Port Hacking, Gunnamatta Bay, NSW, Australia, 34°03'50.0"S 151°08'39.0"E, October 30,1939; QM I.30772, subadult female, Chain Banks, Moreton Bay, QLD Australia, J Johnson, dredge, depth 3–7 m, January 24, 1997; AMS I.12554, adult male, Moreton Bay, QLD Australia, Amateur Fishermans Association of Qld, 1912; CAS 13406, 2 in lot, 13406-1 adult male, 13406-2 juvenile female, Mackay, QLD, Australia, 21°09'48.1"S 149°12'58.2"E, 11 m depth, July 12, 1939; SU 36420, 4 in lot, 36420-1 adult female, 36420-2, adult male, 36420-3 adult female, 36420-4 adult female, 4 miles east of Burnett R, Queensland, Australia, 25°20'21.0"S 151°52'41.7"E, 18 m depth, September 14, 1938; QM I.39230, subadult male, 2 miles NE of Elliot Heads, QLD, 24°55'00.0"S 152°31'00.0"E, March 4, 1982, trawl; QM I. 39656, adult female, east of Waddy Point, 24°58'36.0"S 153°24'08.4"E, March 26, 2005, trawl; CAS-SU 35442, 2 specimens in lot, F43-A adult female, F43-C adult male, Corny Point, South Australia, 34°54'38.7"S 137°03'35.7"E, October 31, 1912; AMS E2914, female holotype, 120 mm, 5–11 km east of Hervey Bay, Fairway Buoy, QLD, Australia, 25°8'59.64"S 152°50'26.94"E, FIS Endeavour, July 7, 1910; AMS IA4205, juvenile female, height 57 mm, Port Curtis, QLD, Australia, 23°55’S 151°23’E, dredged, M Ward & W Boardman, December 14, 1929.

#### Diagnosis.

*Hippocampuswhitei* differs from its congeners by the following combination of characters: trunk rings 11; tail rings 34–35; dorsal fin rays 17–18; pectoral fin rays 16; subdorsal rings three; subdorsal spines four, superior trunk ridge ending with three enlarged spines, superior tail ridge commencing with one enlarged spine (3/0,1,0); cleithral ring spines three, one small spine at each end of pectoral-fin base but none at gill-opening, large single or double spine at ventral extent of head; small lateral head spines, two, one directly posterior of eye, one anterodorsally of operculum and ventral of coronet; distinct snout spine; parietal spine, diminutive or absent; single eye spine, large, protruding dorsally; small single or double spine, rugose, posteroventrally of eye; coronet, distinct and tall, protruding anteriorly in juveniles, angled dorsoposteriorly in adults, five small spines present on apex in a star-like arrangement; superior trunk with enlarged spines on 1^st^ and 8^th^ tail ridges.

#### Redescription.

**Figure 2.**
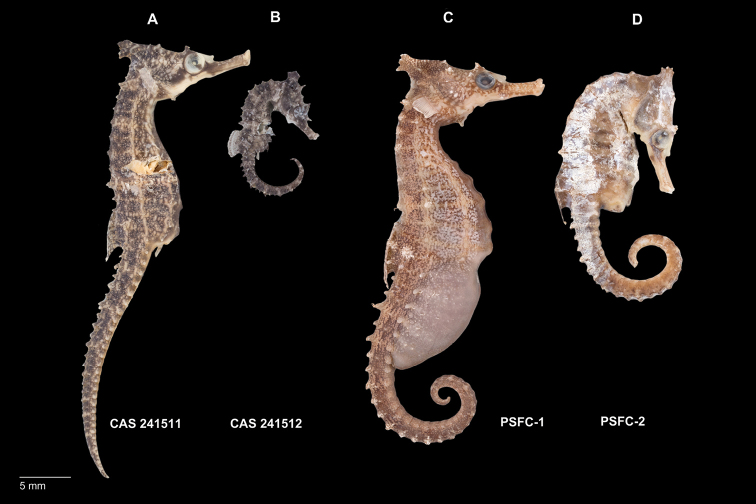
Comparison of non-type specimens of **A***Hippocampusprocerus* CAS 241511, preserved adult female, 142.7 mm SL, Southport, QLD**B***Hippocampusprocerus* CAS 241512, preserved juvenile, 112.7 mm SL, Southport, QLD**1***Hippocampuswhitei* PSFC-DH-1, preserved adult male, 122.1 mm SL, Nelson Bay, NSW**D***Hippocampuswhitei* CAS PSFC-DH-2, preserved subadult female, 47.7 mm SL, Nelson Bay NSW. Note the differences in coronet profile between juvenile/subadult and adult: projecting anteriad in juvenile/subadult versus lower or projecting posteriorly in adults.

**Figure 3.**
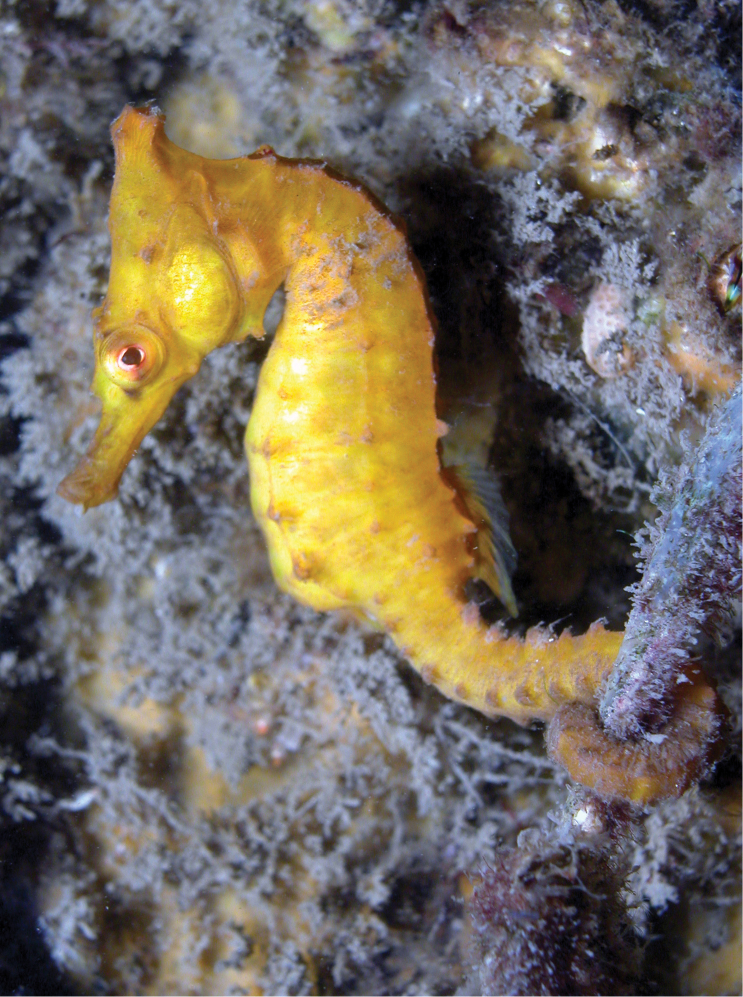
*Hippocampuswhitei* in situ, adult female, Nelson Bay, NSW, Australia at 5 m depth (photograph David Harasti).

**Figure 4.**
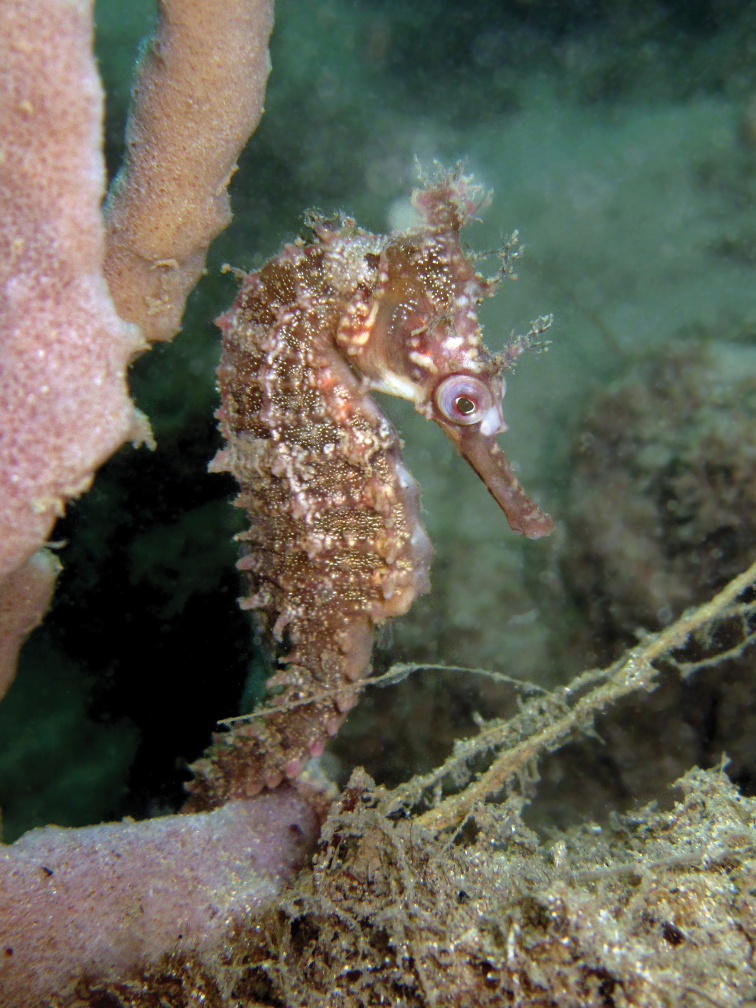
*Hippocampuswhitei* in situ, adult female, Gold Coast, QLD, Australia at 5 m depth (photograph David Harasti).

**Figure 5.**
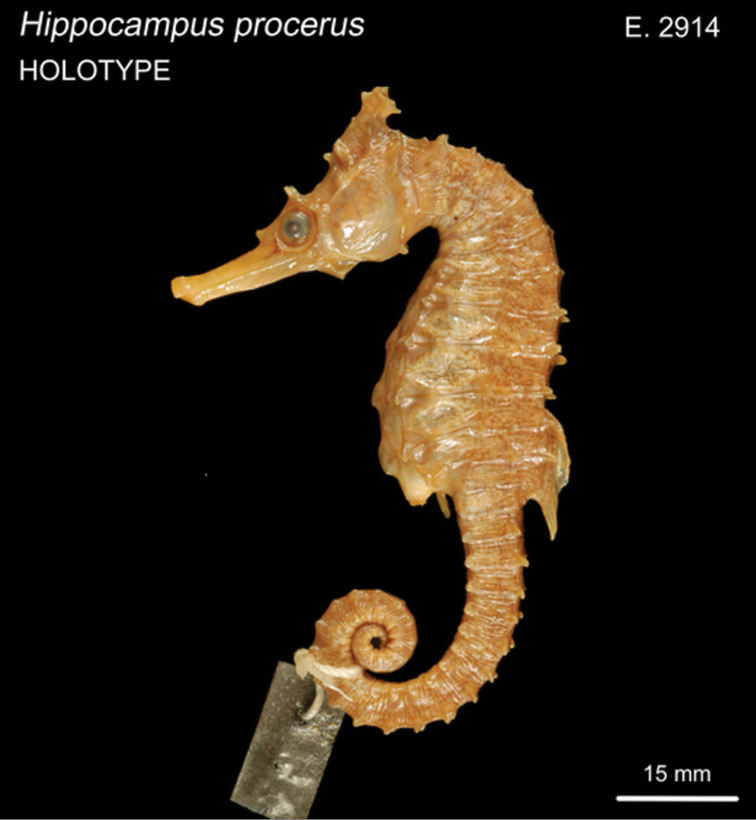
*Hippocampusprocerus*, AMS E2914, adult female, holotype, 120 mm SL, Hervey Bay, Queensland, Australia (photograph Mark Allen).

**Figure 6.**
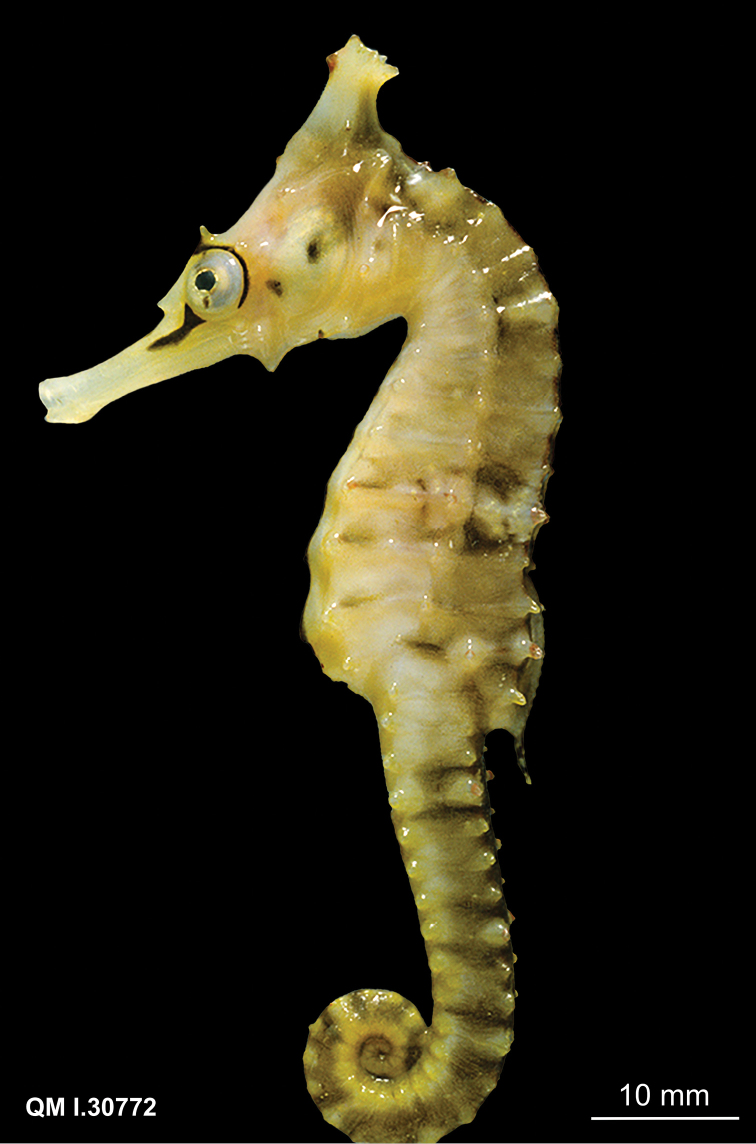
*Hippocampusprocerus*, QM I.30772, subadult female, paratype, 95.0 mm SL, Moreton Bay, Queensland, Australia (photograph Jeff Johnson).

**Figure 7.**
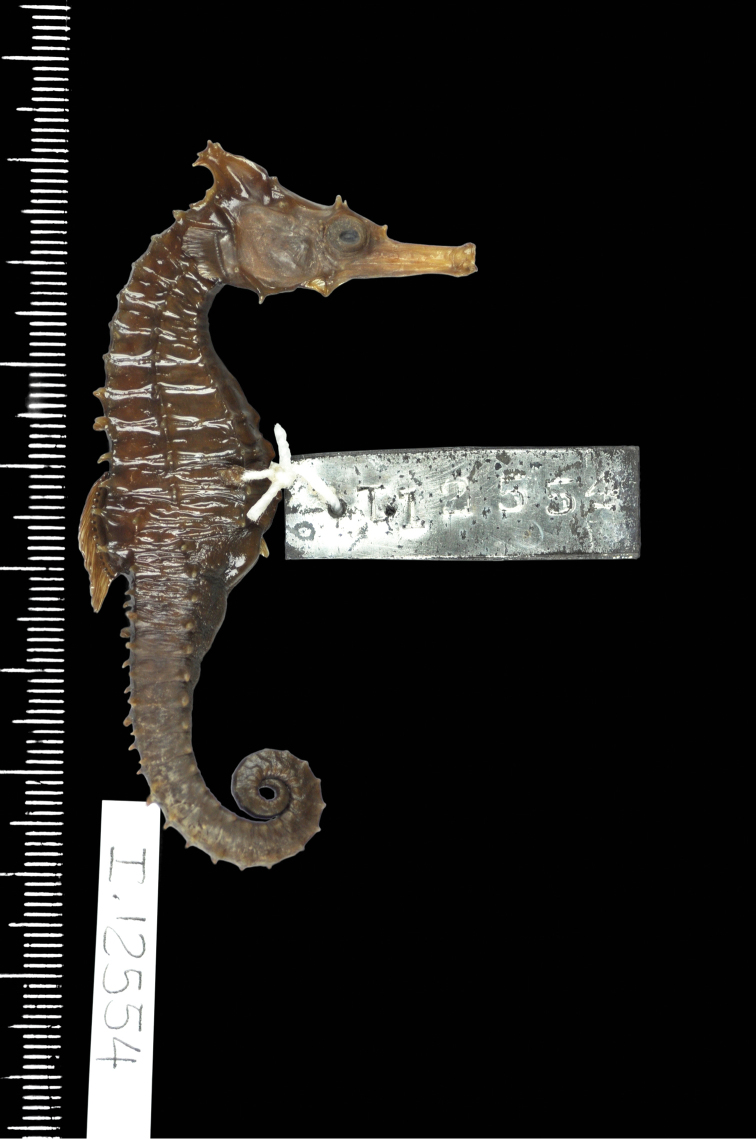
*Hippocampusprocerus*, AMS I.12554, adult male, paratype, 105 mm SL, Moreton Bay, Queensland, Australia (photograph Kerryn Parkinson).

**Figure 8.**
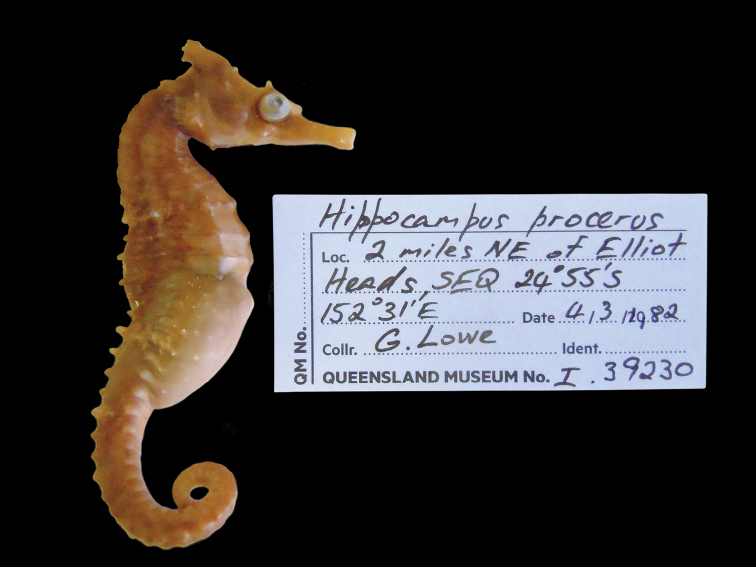
*Hippocampusprocerus*, QM I.39230, adult male, non-type, Elliot Heads, Queensland, Australia (photograph Jeff Johnson).

**Figure 9.**
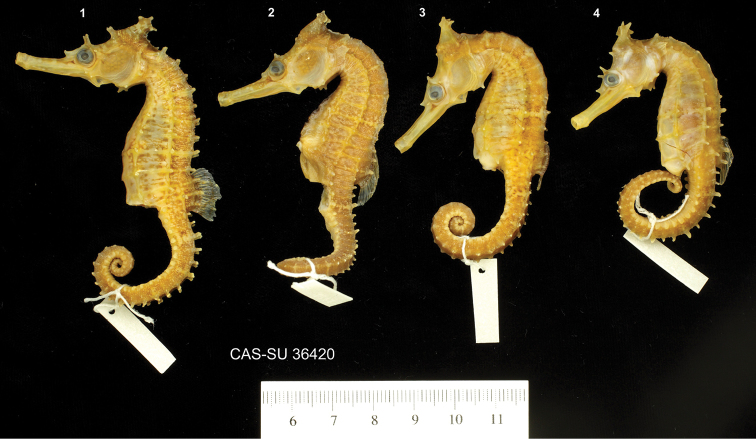
*Hippocampusprocerus*, CAS-SU 36420, 4 specimens in lot, paratypes, Burnett River, Queensland, Australia (photograph Jon Fong).

**Figure 10.**
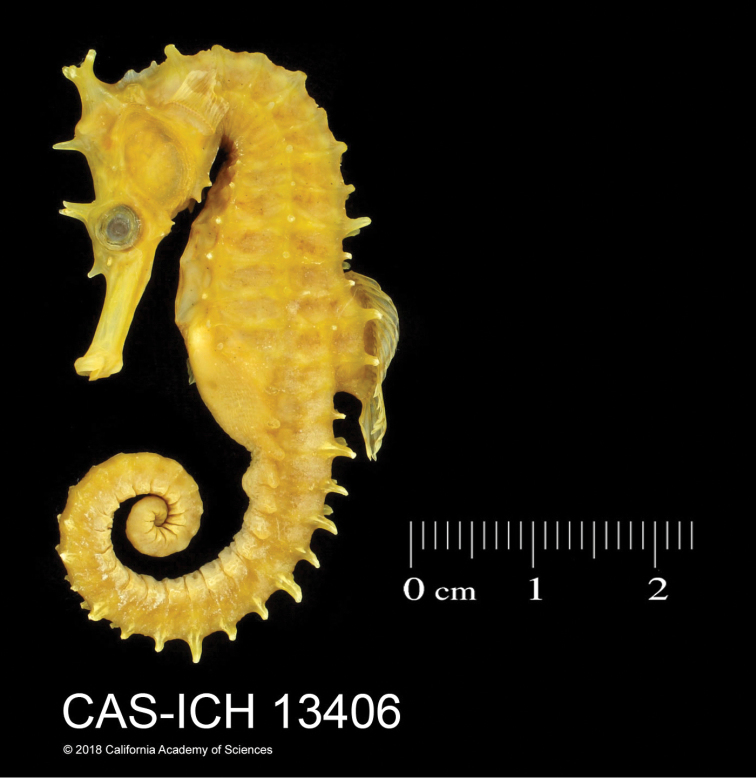
*Hippocampusprocerus*, CAS-ICH 13406, adult male, non-type, 113.4 mm SL, Mackay, Queensland, Australia (photograph Jon Fong).

**Figure 11.**
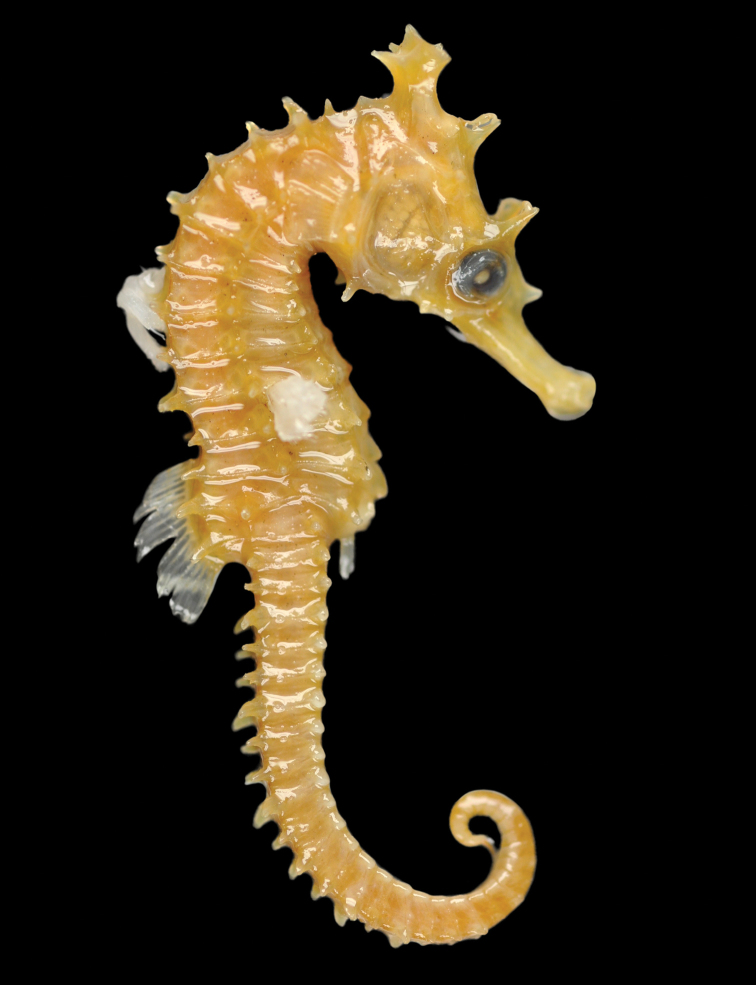
*Hippocampusprocerus*, AMS I.A4205, juvenile female, non-type, Point Curtis, Queensland, Australia (photograph Jeff Johnson).

General body shape as in Figs 2–11. Morphometric and meristic characters are listed in Table 2. Coronet distinct and tall, coronet height 44.8–47.89% in HL, protruding anteriad in juveniles, angled dorsoposteriorly in adults; bilateral gill-openings ventral of coronet; dorsal fin rays 17–18; pectoral fin rays 16; subdorsal rings three; dorsal fin base starting immediately posterior to ninth trunk ring and ending immediately posterior to first tail ring; trunk rings 11; tail rings 34–35. Body spines: coronet with five small spines present on apex in a star-like arrangement; neck spines absent; prominent spine dorsally of eye, small single or double spine, rugose, ventroposteriorly of eye; small lateral head spines, two, one directly posterior of eye, one anterodorsally of operculum and ventral of coronet; cleithral ring spines three, one small spine at each end of pectoral-fin base but none at gill-opening, large single or double spine at ventral extent of head; distinct snout spine on midline between eyes; parietal spine, diminutive or absent in adults, present in juveniles and subadults; subdorsal spines four, superior trunk ridge ending with three enlarged spines, superior tail ridge commencing with one enlarged spine (3/0,1,0); superior trunk with enlarged spines on 1^st^ and 8^th^ tail ridges observed in adults, on 1^st^ , 7^th^, 8^th^, and 9^th^ tail ridges observed in subadults; lateral trunk ridge with small spines on 2^nd^–11^th^ trunk rings; inferior trunk ridge with small spines beginning on 5^th^ trunk ring and ending on 11^th^ trunk ring; superior tail ridge spines well developed anteriorly, becoming smaller posteriorly, with enlarged spines on 1^st^–12^th^ tail rings; inferior tail ridge spines well developed anteriorly, becoming smaller posteriorly, with enlarged spines on 1^st^–8^th^ tail rings; caudal fin absent in juveniles and adults.

#### Morphological remarks.

In his original description, Kuiter (2001:328–329) erected *H.procerus* based on several observations on its distinguishing characters: “Previously confused with *Hippocampustristis* and *H.whitei*, *H.procerus* is more similar to the latter, differing from it in having a taller and spinier coronet, higher fin-ray counts, and generally a spinier physiogamy.” We show that morphometric, meristic, and diagnostic morphological characters reported herein (Tables 2, 3) correspond closely among the non-type specimens of *H.whitei* from NSW, type and non-type specimens referred to as *H.procerus* from Gold Coast Seaway, Moreton Bay, Elliot Heads, Bundaberg, and Mackay, QLD, and the holotype specimen of *H.procerus* from Hervey Bay, QLD.

**Figure 12.**
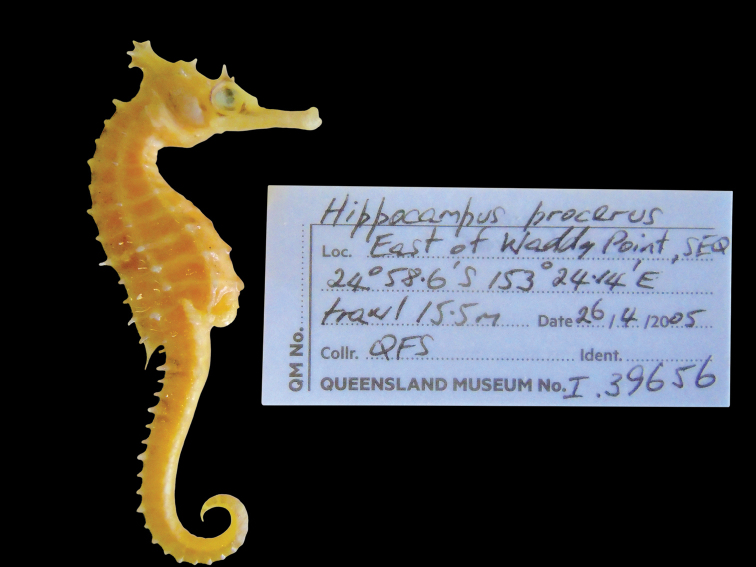
*Hippocampusprocerus*, QM I.39656, adult female, non-type, Waddy Point Queensland, Australia (photograph Jeff Johnson).

Based on the material examined, we found minor variation in coronet height in proportion to the head (45.5–46.6% in *H.whitei* from Nelson Bay, NSW vs. 45.1–47.8% in *H.procerus* from Gold Coast Harbour, QLD, 48.9–52.9% in the paratypes from Moreton Bay, QLD, 44.8% in the holotype from Hervey Bay, QLD, 50.8% in the paratype from Bundaberg, QLD and 46.7% from Mackay, QLD). The non-type specimens are comprised of juveniles, subadults, and adults, all of which exhibit distinct and tall coronets. However, we noted that in juveniles the coronet protrudes anteriad whereas in subadults and adults it is strongly angled dorsoposteriad. Similarly, dorsal fin ray counts exhibited marginal differences (17 in non-type specimens of *H.whitei* vs. 18 in all the specimens of *H.procerus* from Queensland), which may reflect north-south clinal variation. We did not observe an overall spinier physiognomy in the majority of adult specimens of *H.procerus* relative to *H.whitei*. However, a spinier physiognomy was present in one juvenile specimen from Mackay, and Port Curtis, QLD (Figs 10, 11), respectively, and one adult specimen from Burnett River (Fig. 9) and Waddy Point, QLD (Fig. 12), respectively, on all principal trunk and tail ridges and head. It has been observed that in juvenile and subadult *H.whitei* from NSW (<8 cm Total Length) that spines are more pronounced, but as they increase in size the spines disappear, with specimens > 12 cm TL being much smoother and spines not obvious. The adult specimens from Burnett River and Waddy Point, QLD, are an exception to these observations and appear to reflect variation in spine morphology similarly observed in juvenile *H.whitei*.

We also observed across the majority of examined adult specimens the following key diagnostic morphological characters (Table 3): the absence of true neck spines between the coronet and the 1^st^ superior trunk ring spines (small neck spines were detected in juvenile specimens; variation in neck ridge topology is often mistaken for true neck spines in adult specimens), indiscernible or small parietal spine, three cleithral ring spines with the uppermost spine at the dorsal level of the pectoral fin base, presence of a distinct snout spine, four subdorsal ridge spines (3/0,1,0), and superior trunk ridge with enlarged spines on 1^st^ and 8^th^ tail ridges. Based on these findings, we find that spine physiognomy, neck spines present or absent in juveniles and adults, respectively, and subtle differences in meristics, are unreliable diagnostic characters, and that key and informative morphological characters are congruent across all specimens, which conform to the diagnosis of *H.whitei*. Therefore, it can be concluded that the species-level classification of *H.procerus* is unsupported, and that *H.procerus* may be treated as a junior synonym of *H.whitei*.

Several seahorse species endemic to Australia, including Indo-Pacific seahorses with overlapping latitudinal distributions in Queensland, Australia, are superficially similar to and often misidentified as juvenile and adult *H.whitei* (Kuiter 2001; Table 5). These species include *H.abdominalis* Lesson, 1827, *H.angustus* Günther, 1870, *H.breviceps* Peters, 1869, *H.dahli* Ogilby 1908, *H.histrix* Kaup, 1856, *H.jugumus* Kuiter, 2001, *H.kelloggi* Jordan & Snyder, 1901, *H.planifrons* Peters, 1877, *H.spinosissimus* Weber, 1913, and *H.zebra* Whitley, 1964. Despite the morphological similarities, meristic and diagnostic morphological characters support the distinctions among these species (Table 4).

**Table 4.** Comparison of morphological characters in *H whitei* from NSW and other *Hippocampus* spp. occurring in Australia.

	* H. whitei *	* H. abdominalis *	* H. angustus *	* H. breviceps *	* H. dahli *	* H. histrix *	* H. jugumus *	* H. kelloggi *	* H. planifrons *	* H. spinosissimus *	* H. zebra *
Trunk rings	11	12–13	11	11	11	11	12	11	11	11	11
Tail rings	33–35	44–48	39–41	38–42	37–40	33–34	37	39–41	37–38	35–36	37–39
Snout stripe or striation pattern	absent										present
Coronet	distinct, tall	low	distinct, tall	distinct, tall	low	distinct, tall	distinct, tall	distinct, tall	low	distinct, tall	distinct, tall
Subdorsal rings	2+1	3-5+1-2	2+1	3+1	2-3+1-2	2+1	3+2	2+1	3-4+1	2+1	2-3+1
Subdorsal ring spines	3/0,1,0	5/0,0,1,1,1	3/0,1,0	3-4/0,0,1,0	3/0,1,1	3/0,1,0	4/0,1,1,1,1	3/0,1,0	4/0,0,1,1	3/0,1,0	3-4/0,1,0
Cleithral ring	discontinuous										
Upper cleithral spine	dorsal level of pectoral fin base	ventral of gill opening	dorsal level of pectoral fin base	ventral of gill opening	ventral of gill opening	ventral of gill opening	dorsal level of pectoral fin base	ventral of gill opening	ventral of gill opening	ventral of gill opening	dorsal level of pectoral fin base
Neck spine	absent	absent	present	absent	absent	present	present	absent	absent	present	absent
Eye spine	single						double	single			
Lateral head spine	single and small						double and large	single and small			

**Table 5.** Distribution of haplotypes based on partial mtDNA COI sequence data (655 bp) tabulated across sampled *H.whitei* and localities in central NSW and southern QLD.

Species designation	Collection locality	Nucleotide position	Haplotype
Hippocampus whitei_1300a_CO1_Nelson	Nelson	81	A
Hippocampus whitei_1767_CO1_GoldCoast	Gold Coast	174	T
Hippocampus whitei_1783_CO1_GoldCoast	Gold Coast	174	T
Hippocampus whitei_1364_CO1_Forster	Forster	174	T
Hippocampus whitei_1365_CO1_Forster	Forster	174	T
Hippocampus whitei_1295_CO1_Nelson	Nelson	259	C
Hippocampus whitei_1364_CO1_Forster	Forster	342	G
Hippocampus whitei_1365_CO1_Forster	Forster	342	G
Hippocampus whitei_1767_CO1_GoldCoast	Gold Coast	378	A
Hippocampus whitei_1783_CO1_GoldCoast	Gold Coast	378	A
Hippocampus whitei_1364_CO1_Forste	Forster	378	A
Hippocampus whitei_1365_CO1_Forster	Forster	378	A
Hippocampus whitei_1305_CO1_Nelson	Nelson	412	A
Hippocampus whitei_1353_CO1_Nelson	Nelson	412	A
Hippocampus whitei_0418_CO1_Sydney	Sydney	429	G
Hippocampus whitei_0470_CO1_Sydney	Sydney	429	G
Hippocampus whitei_1767_CO1_GoldCoast	Gold Coast	489	G
Hippocampus whitei_1783_CO1_GoldCoast	Gold Coast	489	G
Hippocampus whitei_1364_CO1_Forster	Forster	489	G
Hippocampus whitei_1365_CO1_Forster	Forster	489	G
Hippocampus whitei_1295_CO1_Nelson	Nelson	495	G
Hippocampus whitei_1783_CO1_GoldCoast	Gold Coast	504	T
Hippocampus whitei_1364_CO1_Forster	Forster	513	T

#### Genetic remarks.

Meristic, morphometric, and key diagnostic morphological characters in our study did not support the separation of *H.procerus* from *H.whitei* into two distinct species. Here we further confirm the synonymization of *H.procerus* with *H.whitei* based on partial mitochondrial COI (655 bp) data. This analysis is based on sequences generated from 31 *H.whitei* individuals sampled from Empire Bay, Forster (Wallis Lake), Port Hacking, Nelson Bay, Sydney Harbour, and Tuggerah Lake, NSW, and from 4 specimens referred to as *H.procerus* from Southport, Gold Coast Seaway, QLD. Alignment of sequence data detected 23 variable sites without any indels, resulting in 14 haplotypes: one in Sydney, four in Nelson Bay, five in Forster, and four in Gold Coast Seaway (Suppl. material 1, 2). Three haplotypes are shared between Forster and Gold Coast Seaway whereas no other haplotypes are shared between collection localities. One unique haplotype was obtained in Sydney, four in Nelson Bay, two in Forster, and one in Gold Coast Seaway (Suppl. material 1, 2). Fig. 12 shows a neighbour-joining tree based on the same mtDNA COI data, which recovered *H.procerus* as paraphyletic with respect to *H.whitei*. *Hippocampusprocerus* clustered among individuals of *H.whitei* from Forster, NSW in one subclade and with individuals from several localities in NSW in another subclade. Additionally, genetic distance analysis (uncorrected p distances) of the same data failed to discriminate *H.procerus* from *H.whitei* (Suppl. material 3), which revealed an average intraspecific divergence of 0.002%, further confirming lack of support of species status for *H.procerus*.

**Figure 13.**
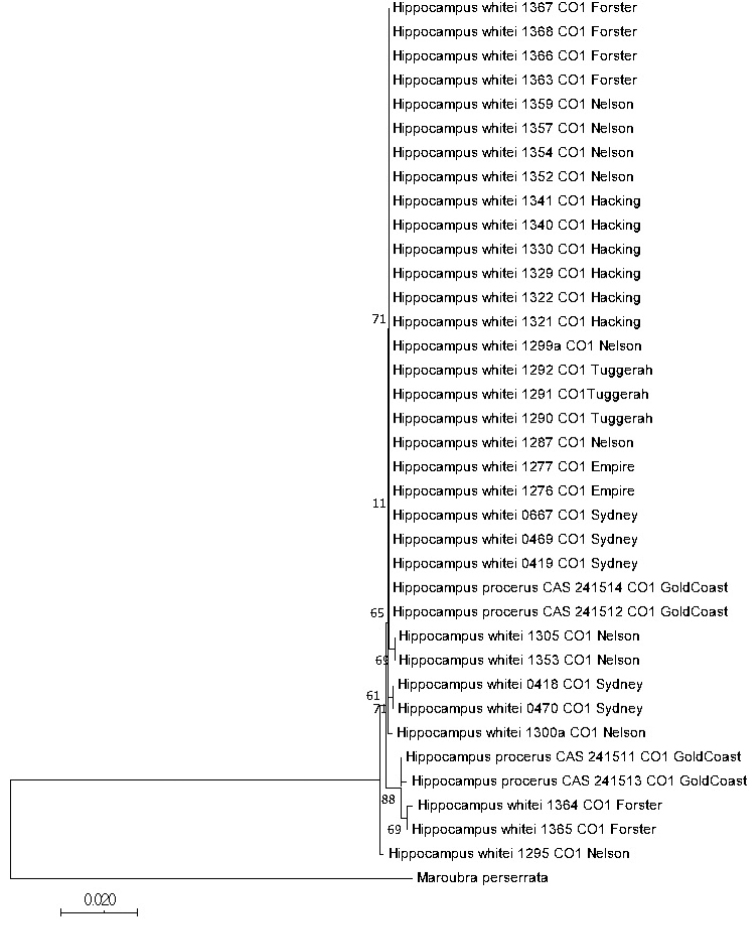
Neighbor-joining tree based on mtDNA COI sequences showing the relationships among specimens of *H.whitei* collected from various sites in NSW and *H.procerus* from Southport, Gold Coast Harbour, QLD. Numbers in branches indicate bootstrap probabilities obtained from 1000 bootstrap replications. Scale bar = genetic distance of 0.02.

#### Distribution and habitat.

*Hippocampuswhitei* is known to occur in coastal estuaries and embayments of central NSW and southern QLD, Australia. In central NSW it has been recorded, from south to north, in St. Georges Basin, Port Hacking, Botany Bay, Sydney Harbour, Hawkesbury River, Tuggerah Lake, Lake Macquarie, Port Stephens, Wallis Lake – Forster, and Tweed River. The record from St Georges Basin was based on a recent sighting and photograph of a small juvenile in January 2018 that was logged through REDMAP (http://www.redmap.org.au/sightings/3379/) and therefore extends the range reported by Harasti et al. (2012) southwards by 70 km. A previous 1903 Australian Museum record from Lake Illawarra cannot be confirmed as the locality information is likely erroneous (Mark McGrouther, pers. comm.) and whilst it is possible that *H.whitei* could occur in Lake Illawarra, at this stage there is no definitive evidence. Museum records indicate the species has been recorded in QLD within the Gold Coast Seaway, at various locations around Moreton Bay estuary, Hervey Bay, Waddy Point, Elliot Heads, Bundaberg, Port Curtis, and Mackay. The synonymization of *H.procerus* extends the northward range significantly by approximately 1,000 kilometres for *H.whitei* to Mackay QLD; as of now, this is the most northern location with confirmed *H.whitei* specimens. However, its current occurrence in the Mackay region, Port Curtis, Burnett River, and Bundaberg, is unknown as it has not been recorded in those locales since 1939, 1929, and 1938, respectively. The most recent northern records are from Elliot Heads in 1982 and Waddy Point in 2005.

Additionally, museum records claim species occurrences of *H.whitei* outside its geographic range, in South Australia, Victoria Australia, Papua New Guinea, South Africa, Solomon Islands, and Vanuatu (Kuiter 2009; Lourie et al. 2016). However, these specimens have subsequently been re-identified by the authors as *H.breviceps, H.camelopardalis, H.kelloggi*, or *H.spinosissimus* (see Table 6). The specimens originally identified as *H.whitei* from Port Moresby, Papua New Guinea and the Solomon Islands are no longer accessible and therefore cannot be re-identified; however, we consider them highly unlikely to be *H.whitei* since it is markedly outside the range for this species. We now consider that the species is constrained to estuaries and embayments along the east coast of Australia from Hervey Bay, QLD, in the north to St Georges Basin, NSW, in the south.

**Table 6.** List of seahorse specimens originally identified as *H.whitei*, including voucher number, collection date, collection location, and status.

Original identification	Voucher number	Collection date	Collection location	Species
* H. whitei *	AMS I.6637	1885	Port Moresby, Papua New Guinea	*Hippocampus* sp.
	SU 35442	1912	Corny Point, South Australia	* H. breviceps *
	AMS IA4205	1929	Port Curtis, Qld, Australia	* H. spinosissimus *
	CAS-SU 31443	1934	Durban Bay, KwaZulu-Natal, South Africa	* H. camelopardalis *
	MNHN-IC-2008-1326	2006	Espiritu Santo, Vanuatu	* H. kelloggi *
	MNHN-IC-2008-1441	2006	Espiritu Santo, Vanuatu	* H. kelloggi *
	MNHN-IC-2008-1662	2006	Malekula, Vanuatu	* H. kelloggi *
	MCZ 168083	unknown	Western Port, Victoria, Australia	* H. breviceps *

*Hippocampuswhitei* occurs in a variety of habitats including seagrasses, soft corals, sponge gardens and artificial structures to depths of 12 m (Hellyer et al. 2011; Harasti et al. 2014a; Manning et al. 2018), and is known to display strong site fidelity and monogamous behaviour (Vincent and Sadler 1995; Vincent et al. 2005; Harasti and Gladstone 2013). The locations with the largest recorded populations are found within Sydney Harbour and Port Stephens (Harasti et al. 2012; Harasti et al. 2014b), beyond which there is very little information about the occurrence, habitat use, and population numbers in QLD as the species is not known to be regularly found in any QLD locations and is seldom seen or collected.

We introduced this paper with a quote from John White (1736–1832) who was under the assumption that the Mediterranean and North Atlantic seahorse *H.hippocampus* and *H.whitei* from Australia were conspecific due to highly similar morphology. Seahorse taxonomy has been in a state of confusion since its inception. While comprehensive revisions of the genus have greatly advanced our understanding of how many species of seahorses exist (Lourie et al. 2016), much further work remains to answer this most fundamental question about one of the world’s most extraordinary fish.

**Video 1.** *Hippocampuswhitei*, in situ, Seahorse Gardens, Nelson Bay, NSW, Australia (video by David Harasti 2011).

## Supplementary Material

XML Treatment for Hippocampus whitei

## Supplementary materials

Supplementary material 1 COI alignnment haplotypes *Hippocampuswhitei*NSWQLD Data type: molecular data This is an open access article distributed under the terms of the Creative Commons Attribution License (CC BY 4.0), which permits unrestricted use, distribution, and reproduction in any medium, provided the original author and source are credited.Graham Short, David Harasti, Healy Hamilton

Supplementary material 2 Distribution haplotypes *H.proceruswhitei* Data type: molecular data Explanation note: Distribution of haplotypes based on partial mtDNA COI sequence data (655 bp) tabulated across sampled *H.procerus* and *H.whitei* from localities in central NSW and southern QLD. This is an open access article distributed under the terms of the Creative Commons Attribution License (CC BY 4.0), which permits unrestricted use, distribution, and reproduction in any medium, provided the original author and source are credited.Graham Short, David Harasti, Healy Hamilton

Supplementary material 3 Genetic analysis uncorrected p distances COI*H.proceruswhitei* Data type: molecular data Explanation note: This document was exported from Numbers. Each table was converted to an Excel worksheet. All other objects on each Numbers sheet were placed on separate worksheets. Please be aware that formula calculations may differ in Excel. This is an open access article distributed under the terms of the Creative Commons Attribution License (CC BY 4.0), which permits unrestricted use, distribution, and reproduction in any medium, provided the original author and source are credited.Graham Short, David Harasti, Healy Hamilton
