# Supplementary figures and images for: Hippocampuswhitei Bleeker, 1855, a senior synonym of the southern Queensland seahorse H.procerus Kuiter, 2001: molecular and morphological evidence (Teleostei, Syngnathidae)
Source: Zookeys. 2019 Feb 14;(824):109–33. doi: 10.3897/zookeys.824.30921 (PMC6389870; doi:10.3897/zookeys.824.30921)

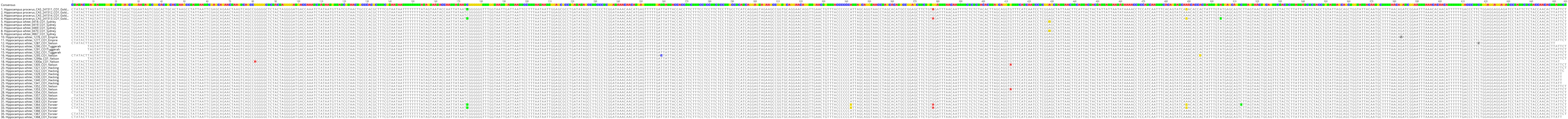

Supplement: Supplementary material 1 [file zookeys-824-109-s001.png]
